# Dental Treatment in Patients with Leukemia

**DOI:** 10.1155/2015/571739

**Published:** 2015-02-15

**Authors:** Caroline Zimmermann, Maria Inês Meurer, Liliane Janete Grando, Joanita Ângela Gonzaga Del Moral, Inês Beatriz da Silva Rath, Silvia Schaefer Tavares

**Affiliations:** ^1^Graduate Program of Dentistry, Federal University of Santa Catarina, 88040-900 Florianópolis, SC, Brazil; ^2^Department of Pathology, Federal University of Santa Catarina, 88040-900 Florianópolis, SC, Brazil; ^3^Stomatology Clinic, University Hospital, Federal University of Santa Catarina, 88040-900 Florianópolis, SC, Brazil; ^4^Hematology Service, University Hospital, Federal University of Santa Catarina, 88040-900 Florianópolis, SC, Brazil; ^5^Department of Dentistry, Federal University of Santa Catarina, 88040-900 Florianópolis, SC, Brazil; ^6^Integrated Multidisciplinary Health, Federal University of Santa Catarina, 88040-900 Florianópolis, SC, Brazil

## Abstract

Dental treatment of patients with leukemia should be planned on the basis of antineoplastic therapy which can be chemotherapy with or without radiotherapy and bone marrow transplantation. Many are the oral manifestations presented by these patients, arising from leukemia and/or treatment. In addition, performing dental procedures at different stages of treatment (before, during, or after) must follow certain protocols in relation to the haematological indices of patients, aimed at maintaining health and contributing to the effectiveness of the results of antineoplastic therapy. Through a literature review, the purpose of this study was to report the hematological abnormalities present in patients with leukemia, trying to correlate them with the feasibility of dental treatment at different stages of the disease. It is concluded in this paper that dental treatment in relation to haematological indices presented by patients with leukemia must follow certain protocols, mainly related to neutrophil and platelet counts, and the presence of the dentist in a multidisciplinary team is required for the health care of this patient.

## 1. Introduction

The insertion of dentistry in the multidisciplinary context of hematology-oncology is an important part of the success of cancer treatment. Oral complications can compromise the protocols of chemotherapy, possibly making it necessary to decrease the administered dose, the change in treatment protocol, or even discontinuation of antineoplastic therapy, directly affecting patient survival [1, 2].

The feasibility to perform certain dental procedures in leukemia patients depends on the overall state of health of the patient, as well as the stage of the disease and/or antineoplastic therapy or hematopoietic stem cell transplantation. Despite the expectation of finding a vast literature on the leukemia/dental relationship, the bibliographic survey conducted (PubMed, BIREME, Journals Portal CAPES, and SciELO) resulted in a few articles involving the amplitude of this relationship. Facing the need to establish protocols for the dental care of oncohematological patients at University Hospital, Federal University of Santa Catarina, a simplified guide for the guidance of residents in dentistry in the evaluation and treatment of these patients was developed. The guide consists of tables correlating phases of chemotherapy and hematopoietic stem cell transplantation to the most common dental procedures (classification adapted from Sonis et al. [3]).

## 2. General Considerations regarding Leukemia

Leukemia is a malignant disease of the blood, where the uncontrolled proliferation of immature blood cells that originate from hematopoietic stem cell mutation occurs. Eventually these aberrant cells compete with normal cells for space in the bone marrow, causing bone marrow failure and death [4].

### 2.1. Classification

The most common leukemias are generally classified as (1) acute lymphocytic, (2) acute myeloid, (3) chronic lymphocytic, and (4) chronic myeloid. The classification criteria of leukemia is histological and is based on (a) the similarity between the leukemic cells and normal cells (myeloid versus lymphoid) and (b) the clinical course of the disease (acute versus chronic) [4].

The acute forms of leukemia result from the accumulation of immature and functionless cells in the bone marrow, with rapid progression [5], rapidly fatal in untreated patients [6]. Chronic leukemias, in turn, begin slow with uncontrolled proliferation of more mature and differentiated cells [5].

### 2.2. Treatment

The treatment of leukemia depends on factors such as type and subtype of the disease, risk factors, and age of the patient. In general, the recommended treatment is chemotherapy with or without adjuvant treatments. Hematopoietic stem cell transplantation (HSCT) is performed, in general, in the acute forms of the disease and some cases of chronic myeloid leukemia:

- acute lymphoblastic leukemia (ALL): prophase (initial reduction of leukemic cells), induction (achieve complete remission), consolidation (increase the quality of remission), intensification (postremission further reduction), and maintenance therapy (maintenance of consolidation); prophylactic central nervous system (CNS) therapeutic irradiation or irradiation if CNS is involved; the HSCT can be done in some cases [7];
- acute myeloid leukemia (AML): induction (until complete remission), consolidation, and intensification [8];
- chronic myeloid leukemia (CML): remission of leukemic cells and Philadelphia chromosome-positive with high doses of chemotherapy, monitoring of therapy, and HSCT [9];
- chronic lymphocytic leukemia (CLL): conventional treatment is not curative; chemotherapy is performed as a control [10].

#### 2.2.1. Special Considerations about HSCT

Treatment with HSCT aims to repopulate the marrow, previously destroyed with high doses of chemotherapy with or without radiation, for normal healthy cells. The HSCT can be of the autologous type (patient's own hematopoietic stem cells) or allogeneic (hematopoietic cells obtained from a donor) [4, 11] and consists of five phases: (1) preconditioning, (2) neutropenic phase conditioning, (3) engraftment to hematopoietic recovery, (4) immune reconstitution/recovery from systemic toxicity, and (5) long-term survival [1].

The main complications of HSCT are graft rejection (for failure in the patient's immunosuppression) and the graft-versus-host disease (GVHD), where immunocompetent donor cells attack the patient's antigens, which may lead to the depletion of T lymphocytes. Potentially fatal, GVHD can occur soon after HSCT (acute GVHD) or after a few months (chronic GVHD or cGVHD). With deep and long immunosuppression, the patient becomes susceptible to fungal and viral infections [4].

### 2.3. Oral Manifestations of Leukemia

In acute leukemias, gingival hyperplasia is generally observed, localized or generalized, mainly affecting the interdental papillae and the marginal gingiva caused by inflammation, or leukemic infiltration, and may be localized or generalized, the latter being the most frequent form [3, 5]. The infiltration of leukemic cells may also involve periapical tissues and simulate, both clinically and radiographically, periapical inflammatory lesions [6]. In chronic leukemia, the leukemic infiltrates in oral tissues is less frequent and can be observed: pallor of the mucosa, soft tissue infections, and generalized lymphadenopathy [5].

The manifestations of thrombocytopenia are more common when the platelet count is below 50,000 cells/mm^3^ [12] and may manifest as bruising, petechiae in the hard and soft palate, and also spontaneous gingival bleeding, especially if the platelet count is below 20,000 cells/mm^3^ [6].

Opportunistic infections with* Candida albicans* and Herpes viruses are common and can involve any area of the mucosa. Ulcers can also result from impaired immune defense in combating normal microbial flora [6].

### 2.4. Oral Manifestations Related to HSCT

The most common oral manifestations related to pre-, immediate post-, and late post-HSCT are summarized in Table 6.

The oral manifestations that may be present are correlated with the phases of HSCT [1]: (1) preconditioning: oral infections, ulceration, bleeding, and temporomandibular joint dysfunction; (2) neutropenic phase conditioning: mucositis, dysgeusia, xerostomia, bleeding, oral pain, opportunistic infections, neurotoxicity, and temporomandibular dysfunction, usually manifesting with high prevalence and severe forms; at this stage, the patient may develop hyperacute GVHD with further severe oral complications; (3) engraftment to hematopoietic recovery: opportunistic infections are common and acute GVHD becomes a concern; bleeding may be present, xerostomia, neurotoxicity, granulomas/papillomas, and temporomandibular dysfunction; (4) immune reconstitution/recovery from systemic toxicity: salivary dysfunction, late viral infections, craniofacial growth abnormalities, cGVHD, and squamous cell carcinoma; and (5) the long-term survival: in pediatric patients, particularly children under 6 years, one can observe complications in the development of bones and teeth; at this stage, recurrence and malignant neoplasms can be observed.

In the occurrence of GVHD, mucositis, gingivitis, erythema, and pain are usually observed. In cGVHD, the most common oral manifestations are lichen-type features, hyperkeratotic plaques, mucocele, atrophic mucosa, ulceration [13, 14], fibrosis with limited mouth opening, hyposalivation, and xerostomia [13–16]. In addition, secondary to cGVHD, the patients have a greater tendency to develop malignancies [14, 17, 18].

## 3. General Considerations regarding Dental Treatment

The dental management of patients with leukemia is necessarily embedded in a multidisciplinary context, because the medical complexity that this patient presents may interfere in the determination of priorities and the time available for dental treatment. For the US National Cancer Institute [2], the multidisciplinary team should have oncologists, nurses, dentists (general and stomatological practitioners), social workers, nutritionists, and other health professionals, which may contribute to the prevention and treatment of oral complications in these patients.

Sonis et al. [3] proposed the classification of patients into categories of high, moderate, and low risk for dental treatment, based on the type of leukemia (acute or chronic) and chemotherapy. Patients at high-risk are those with active leukemia, which have a high number of neoplastic cells in the bone marrow and peripheral blood; because of this, they are thrombocytopenic and neutropenic. This risk group also includes antileukemic patients under treatment, and as a result of therapy, present bone marrow suppression. Considered moderate risk patients are those who successfully completed the first phase of treatment (induction) and are undergoing the maintenance phase, thus not showing signs of malignancy in the bone marrow or peripheral blood; however, they present myelosuppression due to chemotherapy. In the low-risk category are patients who successfully completed treatment and present no evidence of malignancy or myelosuppression.

Basic health care should be part of the patient's routine during antineoplastic therapy and HSCT for maintaining good oral health and reducing the risk of systemic infections of oral origin. The objectives of care include prevention of infection, pain control, maintenance of oral functions, and management of complications of antineoplastic therapy, aimed at improving the quality of life of patients [19].

Little et al. [5] and Elad et al. [19] reinforce that the role of the dentist should occur at three different moments:

1. pre-antineoplastic treatment evaluation and preparation of patients for this,
2. guidelines and oral health care during treatment,
3. posttreatment care.

### 3.1. Pre-Antineoplastic Treatment Assessment and Patient Preparation

Dental treatment at this stage is based on priorities and should be directed to the acute needs; elective treatment can be postponed to a time when the patient is appropriate for clinical and laboratory conditions [1, 2, 5, 19, 20].

The dental examination, if possible, should occur immediately after diagnosis and before initiation of chemotherapy so as to permit the removal of sources of infection of dental origin [3, 5, 20, 21], since expected neutropenia during chemotherapy predisposes patients to the spread of infection [5].

The objectives of the pre-antineoplastic treatment dental evaluation are as follows [1, 3, 5, 20–22]:

1. identify and eliminate sources of existing or potential infection, without, however, promoting complications or delaying cancer therapy;
2. educate the patient (or their relatives) about the importance of maintaining oral health in reducing problems and oral discomfort before, during, and after cancer treatment;
3. warn about the possible effects of antineoplastic therapy in the oral cavity, such as mucositis;
4. identify specific issues of the diagnosis of leukemia, such as leukemic infiltrates in oral tissues.

Injury prevention and oral infections is the focus of dental treatment in leukemic patients and the care with oral hygiene (brushing, use of fluoride, and noncariogenic diet) should be emphasized throughout treatment [1, 5, 19].

Data from the US National Cancer Institute [2] allege that some cancer centers encourage tooth brushing and flossing, while others indicate the interruption of brushing and flossing when blood components have a drop below specified limits (e.g., platelets <30,000 cells/mm^3^). However, according to the institute itself, there is no evidence in the literature regarding the best approach. The centers providing strategy argue that the benefits of proper brushing and proper flossing outweigh the risks, because the interruption of routine oral hygiene increases the risk of infection, and this could promote bleeding as well as increase the risk of local and systemic infection. Elad et al. [23] agreed that dental treatment prior to HSCT is preferred to no dental intervention.

### 3.2. Oral Health Care during Antineoplastic Treatment

Patients undergoing chemotherapy have become immunosuppressed and therefore susceptible to systemic infections. They are classified as high-risk patients, not only by the possibility of developing infection, but the extent and severity of this potential, which can have quick course and be potentially fatal [24].

The objectives of dental care during chemotherapy are as follows [1]:

1. maintain optimal oral health;
2. treat side effects of antineoplastic therapy;
3. reinforce to the patient the importance of oral health in reducing problems/discomforts arising from chemotherapy.

Apart from oral mucositis, the main oral complication of chemotherapy, other changes may occur, such as bleeding, increased rates of caries, infections (bacterial, viral, or fungal), gingival abscesses, recurrent herpetic stomatitis, candidiasis, salivary gland dysfunction, xerostomia, dysgeusia, and pain [2, 3, 20, 24]. It is important to realize that infections in the oral cavity can progress to systemic infections, worsening the health status of the patient, and the presence of a dentist and/or stomatologist provides important support to the medical staff [2, 3, 21, 25, 26].

### 3.3. Post-Antineoplastic Treatment Oral Health Care

In the post-antineoplastic treatment phase, patients are considered cured of leukemia and not having oral manifestations due to illness or chemotherapy, with the exception of those with sequelae of radiotherapy or children who received chemotherapy in the stage of tooth formation [3], which may present hypoplastic areas on tooth enamel (mineralization disorder) and changes in the development of dental roots (which are presented short and V-shaped) [27].

### 3.4. Special Considerations about Oral Health in HSCT Patients

Considerations regarding the oral health in HSCT patients in pre-, immediate post-, and late post-HSCT are summarized in Table 6.

The principles of dental care before HSCT are very similar to those discussed in Section 3.1 and must consider the following features: (1) In HSCT, the total dose of chemotherapy and/or irradiation of the body is performed a few days before transplantation and (2) immunosuppression will be long term after transplantation [1].

Even though common oral diseases such as periodontal disease can impact systemically in HSCT patients, the pre-HSCT assessment by a dentist is needed, and should include maintenance of the oral health guidelines.

All patients undergoing HSCT should receive specific care, particularly those who develop cGVHD. A complete dental evaluation should occur regularly, and special attention should be focused on early detection of oral cancer and precursor lesions [14, 17, 18, 28]; diagnosis and treatment of mucosal lesions [14, 28] and erythema or lichen-type features with symptomatology [18]; caries prevention [14, 28, 29]; reestablishment of oral health in case of rampant caries [14, 30], with the possibility of use of fluoride applications [14] or silver diamine fluoride for disease control and relief of hypersensitivity [30]; and pharmacological treatment [14, 28, 31] or nonpharmacological treatment [14, 28, 29] of hyposalivation and xerostomia.

The diagnosis of oral cGVHD depends on the patient's history, clinical findings, and early signs and symptoms [14] and it is generally not necessary to perform a biopsy [28].

Even after immunosuppressive therapy, patients who develop cGVHD require long term intensive care. In the care are the reduction of symptoms, resolution of painful injuries, and prevention and management of secondary complications, as well as guidelines for the maintenance of good oral hygiene [14].

## 4. Dental Procedures in Different Stages of the Disease and Treatment

Dental treatment should be planned according to the antineoplastic therapy [3] and HSCT [19]. The execution of some dental procedures—especially those of invasive character—depends on the overall health status of the patient and stage of antineoplastic treatment in which it lies. Considering the risk of bleeding and serious infections associated with invasive procedures in the oral cavity, there are already some protocols that emphasize the importance of evaluating certain hematological indices, mainly neutrophils and platelets. Variation was observed among authors regarding the amounts considered minimal for invasive dental procedures in the pre- and transchemotherapy phases. Tables 1 and 2 show the variation as well as meeting the recommendations regarding the need for transfusions, antibiotic prophylaxis, and postponement of dental treatment [1–3, 5, 22, 32–34].

These explained variations will be presented and discussed in Tables 3, 4, and 5, constructed for each phase of treatment.

### 4.1. Dental Treatment in the Prechemotherapy Phase

Table 3 (prechemotherapy) summarizes the dental procedures and their limitations in the literature, concerning hematological indices and necessary precedence for the procedure considering the initiation of chemotherapy.

Initially, dental treatment should be directed to the acute needs [5]. Elective treatments should be postponed to an opportune time when the patient is in good clinical and hematological conditions [1, 2, 19, 20].

The US National Cancer Institute [2] argues that interventions at this stage should be directed to the treatment of lesions in the oral mucosa, carious and endodontic lesions, periodontal disease, poorly fitting dentures, orthodontic appliances, temporomandibular joint changes, and salivary dysfunction.

Elad et al. [19] recommend that the dentist should eliminate potential sources of trauma in the mucosa, such as orthodontic appliances, ill-fitting dentures, unsatisfactory restorations, traumatized teeth, and dental calculus. They also claim that nonrestorable teeth (with root exposure, severe periodontal involvement and impacted with pericoronitis signals) must be extracted. In the case of restorable teeth, it should be determined if there is enough time for proper treatment. Multiple extractions should be considered if teeth are neglected by the patient.

According to Sonis et al. [3], decayed teeth should be restored when there is no risk of pulpal involvement; if this risk exists, they should be removed or treated endodontically. They also state that any tooth with questionable prognosis should be removed, as well as teeth with periodontal involvement and partially erupted third molars which may prove to be the foci of pericoronitis.

The American Academy of Pediatric Dentistry [1] argues that when all dental needs cannot be addressed before the start of cancer therapy, priority should be eliminating sources of infection and trauma, as well as extractions and periodontal care. Endodontic treatment of symptomatic nonvital teeth should be done at least a week before the start of chemotherapy in order to have sufficient time to evaluate the success of treatment; if this is not possible, extraction is indicated. Teeth that cannot receive endodontic treatment in one session also have extraction as a treatment of choice, with antibiotic prophylaxis (penicillin or clindamycin) for about a week. In asymptomatic teeth, endodontic treatment should be delayed until the haematological indices of the patient stabilize (this includes endodontically treated teeth with periapical lesions, without signs and symptoms of infection). Teeth unable to be restored with periodontal pockets greater than 6 mm, with acute symptomatic infection, significant bone loss, furcation exposure, mobility, and impacted and residual roots should be removed. Ideally, extraction should occur two weeks before the start of the antineoplastic treatment or at least 7 to 10 days before. Finally, the academy recommends that surgical procedures should be as atraumatic as possible, without leaving remnant bone edges and with satisfactory suture of the wound. If there is infection associated with the tooth, antibiotic prophylaxis should be done for a week and by the drug ideally chosen by antibiogram.

According to Little et al. [5], the extraction should be made, preferably three weeks prior to chemotherapy or radiotherapy and at least 10 to 14 days earlier. If the platelet count is less than 50,000 cells/mm^3^, invasive procedures should be avoided; when less than 40,000 cells/mm^3^, this indicates performing transfusions. Antimicrobial prophylaxis is recommended when the leukocytes count is less than 2,000 cells/mm^3^ or less than 500 cells/mm^3^. Partially erupted molars can be a source of infection due to pericoronitis. If the gingival tissue which partially covers the tooth is a potential factor for infection, the tissue should be excised, if the hematological levels permit.

Toljanic et al. [35] conducted a prospective study aimed at assessing a minimum protocol for prechemotherapy dental treatment involving 48 patients with solid or hematological neoplasms, empirically classifying the chronic changes of odontogenic origin as mild, moderate, or severe, considering the probability of them developing into acute processes during chemotherapy. In acute diagnosed alterations based on signs (edema, purulent drainage, and compatible radiographic changes) and symptoms (pain, tenderness, and fever), the source of infection was removed before chemotherapy. In contrast, in chronic changes, the source of infection was not removed. Chronic lesions of odontogenic origin were identified in 79% of patients, where 44% were considered serious chronic illness; of these, only 4% had episodes of fever diagnosed as odontogenic in origin, which were treated with antibiotics without interruption of chemotherapy. For the authors, these results demonstrated that patients with chronic odontogenic lesions can safely undergo chemotherapy without dental procedures, since the conversion of chronic processes in acute cases was uncommon and when sharpening occurred it was effectively treated without interruption of therapy and without adversely affecting the oncological treatment. This strategy would significantly change the established protocols, which recommend a more aggressive prechemotherapy dental treatment. The authors reasoned that, depending on the severity of the cancer, there may be a need to quickly start chemotherapy to maximize its therapeutic effects and in that narrow window of time, the extraction of teeth without potential recovery may be the only viable treatment option and still the possibility of infection after tooth extraction would delay the repair of the wound. It is concluded that the treatment of chronic odontogenic lesions can be safely postponed until the end of chemotherapy, considering the therapeutic benefits.

According to Haytac et al. [32] a neutrophil count of 1,500/mm^3^ and platelets of 40,000 cells/mm^3^ are required for performing periodontal probing or extractions. The procedures must be performed under antibiotic cover and at least three days before the start of chemotherapy (approximately 10 days before the granulocyte count falls below 500 cells/mm^3^); when not possible, dental treatment should be postponed until the haematological indices increase.

The American Academy of Pediatric Dentistry [1] argues that orthodontic appliances should be removed if the patient has deficient oral hygiene and/or in cases where the protocol of antineoplastic treatment confers risk for developing moderate or severe oral mucositis; simple devices that do not irritate the soft tissues, removable appliances, or retainers well adapted may be maintained provided that the patient has good oral hygiene. Sheller and Williams [36] defend that orthodontic appliances should be removed.

It is important that the dentist is aware of the signs and symptoms of periodontal disease, since these can be subtle when the patient is immunosuppressed [1, 37].

After treatment of acute needs, other procedures such as smoothing of rough restorations, rounding, or restoration of tooth fractures may be performed, in addition to the assessment of dentures. Scaling procedures and root planning should be performed to prevent periodontal infections, as well as enhancing oral hygiene instruction and the use of mouthwash with fluoride in preventing dental caries [3, 5].

### 4.2. Dental Treatment in the Transchemotherapy Phase

Table 4 refers to the stage where the patient is undergoing chemotherapy and lists the dental procedures and restrictions assigned to it, referring to haematological indices and considering the period between cycles of chemotherapy.

In high-risk patients (active or under leukemia bone marrow suppression) dental intervention is limited to emergency care. However, oral hygiene must be maintained by the use of mouthwashes and mild antimicrobial and antiseptic solutions, in order to promote ulcer healing and minimize complications from infection. When there is evidence of oral infection, high-risk patients should receive broad-spectrum antibiotics intravenously [3, 5]. In UH/UFSC 0.12%, solution-based nonalcoholic chlorhexidine gluconate is used in the form of daily mouthwash or applied with gauze or swab.

In patients at moderate risk (maintenance phase), the myelosuppression peak is most evident, usually after 14 days of drug administration, and at this time, dental treatment should be avoided; before or 21 days after the start of chemotherapy the treatment can be performed; however, the doctor should be consulted. If the leucocyte count is below 3,500 cells/mm^3^ or the platelet count is less than 100,000 cells/mm^3^, elective dental treatment should be postponed [3]. According to these authors, type I procedures can be performed according to standard protocols, since in types II, III, and IV procedures, antimicrobial prophylaxis is recommended.

Tong and Rothwell [24] do not recommend routine antibiotic prophylaxis for dental procedures in patients undergoing chemotherapy; however, for invasive procedures such as tooth extractions and other deep periodontal scaling procedures that can cause significant bleeding and propagation of bacteria into the bloodstream, antibiotic coverage should be performed.

Koulocheris et al. [34], citing other authors, state that in oral surgical procedures during chemotherapy, the benefit/risk to the patient must be considered, as well as the consequences of chemotherapy cycles; these procedures should therefore be planned and agreed on an interdisciplinary level. Furthermore, the surgical procedure should be the most conservative possible, with trans and postantibiotic prophylaxis and postoperative platelet transfusion if necessary. It is claimed, in addition, that an absolute neutrophil count greater than 1000 cells/mm^3^ and platelet count of at least 60,000 cells/mm^3^ are acceptable rates for oral surgeries.

When there is spontaneous bleeding resulting from minor trauma, the dentist should strive to improve the oral hygiene of the patient and use local measures to control the bleeding. If these measures are not sufficient, platelet transfusion may be required [5].

The management for control of oral bleeding includes the use of vasoconstrictor agents, clots, and tissue guards. To reduce the flow of blood from bleeding vessels, one can use epinephrine; to organize and stabilize blood clots, topical thrombin and/or collagen hemostatic agents can be used; and to stanch the bleeding sites and protect organized clots, the application of the mucosa adhesive products, such as those based on cyanoacrylate, may be performed. The topical aminocaproic acid can be useful in patients with friable clots and intravenous administration may be considered, in some cases, to improve coagulation and the formation of stable clots [2]. Topical use of tranexamic acid is also cited as an effective hemostatic in reducing the incidence of postoperative bleeding in patients taking continuous use of oral anticoagulants [38, 39]. Coetzee [40] reports the empirical use of 500 mg crushed tablets ground in moist cotton at the site of the surgical wound after tooth extraction, or diluted in water for mouthwash, suggesting it as an option.

### 4.3. Dental Treatment after Chemotherapy

Table 5 relates the postchemotherapy period and summarizes the considerations and constraints in the literature for performing dental procedures.

Patients who were cured of leukemia are considered to be of low risk and can be met with normal dental treatment regimens [3]. After completion of cancer therapy and only after two years free of disease, the orthodontic treatment that was interrupted can be restarted [36].

Koulocheris et al. [34] suggest that antibiotic prophylaxis during oral and maxillofacial surgical procedures should be performed for at least six months after the completion of chemotherapy.

### 4.4. Dental Treatment in Different Phases of Chemotherapy Treatment

Table 7 shows, in summary form, the considerations and limitations related to dental procedures at different stages of antineoplastic treatment.

Noninvasive procedures do not require additional care and may be performed at any stage of the disease or chemotherapy. Fit in this situation the type I (clinical examination, radiographs, and oral hygiene instruction) and type II procedures (simple restorations, atraumatic restorative treatment—ART, supragingival scaling and prophylaxis) [3]. Since the priority is the treatment of leukemia before the diagnosis (prechemotherapy phase) or during antineoplastic treatment, some dental procedures, classified as type I (molding) and type II (orthodontic treatment), are considered elective for these patients, and even in the postchemotherapy phase, some restrictions must be considered when related to orthodontic treatment.

There are procedures, however, that are considered nonsurgical (type III)—such as the realization of more complex restorations, scaling, root planning (subgingival), and endodontic treatment—but they require special care in the prechemotherapy and transchemotherapy phases, considering the general state of health and the risk versus benefit to the patient. Some authors suggest that periodontal procedures such as probing and periodontal scaling could cause bacteremia [41–44]. In addition, the endodontic treatment protocol for asymptomatic teeth, according to the literature, is not well established. Thus, the realization of endodontics has to justify the removal of infectious foci; however, some professionals prefer, in such situations, to adopt a radical behavior, performing the extraction of the dental element in question in order to avoid future complications. In the postchemotherapy period, these dental procedures can be performed without restrictions.

Invasive procedures such as simple extractions (type IV), multiple extractions (type V), or those of an entire arch or entire mouth (type VI) can be performed, but its execution is dependent on the patient's risk and should therefore the risk versus the benefit should be considered in specific situations [3]. We believe that gingivoplasty procedures, multiple extractions (no infectious foci) flap surgery (gingivectomy), extraction of single or multiple impacted teeth, apicoectomy, placing implants, and orthognathic surgery are considered elective treatments before the diagnosis and/or treatment of leukemia and should not be performed until the patient completes—and maintains—their antineoplastic treatment successfully.

### 4.5. Special Considerations about Dental Treatment in HSCT Patients

The considerations of the dental treatment in the pre-, immediate post-, and late post-HSCT are summarized in Table 6.

The American Academy of Pediatric Dentistry [1] recommends that dental treatment is made dependent on each phase of HSCT. In the preconditioning phase, all dental treatment should be completed before the patient becomes immunosuppressive. Elective treatment should be delayed until the reestablishment of immunity (at least 100 days after transplant, or more in the case of oral complications or other cGVHDs). In the neutropenic conditioning phase, the focus is the monitoring and management of oral complications, with reinforcement of maintenance guidelines of good oral hygiene. Dental procedures should not be performed at this stage; in the case of emergencies, dental approach should be developed with the participation of the medical staff. In the engraftment phase to hematopoietic recovery, a dental assessment should be performed, with special attention to xerostomia and GVHD. Invasive procedures should be made only with the approval of the medical staff; the patient should be encouraged to maintain good hygiene with a noncariogenic diet. In the immune reconstitution/recovery phase from systemic toxicity, a periodic evaluation with dental radiography can be performed; however, invasive procedures should still be avoided; clarifying the risks and benefits of the use of orthodontic appliances is recommended. Finally, in the long-term survival phase, a routine dental evaluation with interdisciplinary and multidisciplinary involvement is necessary.

The authors differ somewhat as to the best approach for a better dental protocol in HSCT patients, but are unanimous in stating that the assessment and dental care are needed.

Raber-Durlacher et al. [37] investigated the correlation between gingivitis/periodontitis and the development of bacteremia during the period of neutropenia after HSCT. Eighteen patients were examined and classified into two groups: (1) periodontally healthy (probing pocket depth: PPD ≤ 4 mm and bleeding on probing: BOP ≤ 10%) and (2) the presence of gingivitis (PPD ≤ 4 mm and BOP > 10%) or periodontitis (PPD > 4 mm and BOP ≥ 10%). Only 28% of the patients were considered periodontally healthy. Of the total, 67% of the patients developed bacteremia (diagnosed by blood samples collected 2 times per week), and group 2 had more frequent episodes during the neutropenia phase than group 1. The authors suggested that gingivitis and periodontitis may represent a risk factor for the development of bacteremia, which has also been shown in other studies [43, 44]. They further stated that the exacerbation of gingivitis and chronic periodontitis is rare, probably due to the institution of prophylactic therapy; on the other hand, common illnesses should not be overlooked, such as potential underdiagnosed of bacteremia source, particularly during periods of neutropenia.

Melkos et al. [45] conducted a prospective study of 58 patients undergoing HSCT and evaluated the preexisting odontogenic lesions, dental care, and the effect of both on the medical procedure. All patients were referred for a dental evaluation before the HSCT, being examined by two experienced dentists through clinical examination (soft and hard tissues) and radiographic (panoramic and occasionally for symptomatic periapical teeth). Infectious foci teeth were considered with periapical and periodontal infection and those semi-impacted. The type of pretransplant dental work and the occurrence of posttransplant complications (mucositis, infections, graft versus host disease (GVHD), and relapse of disease) were evaluated for an average of 50.45 weeks after the date of transplantation. The protocol for dental treatment included restoration of active caries and extraction of nonrestorable teeth and those with advanced periodontal disease; nonvital teeth were endodontically treated or extracted, whereas periapical lesions were treated endodontically, performing apicoectomy or extraction. Patients were divided into two groups: (I) no infectious foci or complete dental treatment before transplantation (*n* = 36) and (II) with infectious foci, submitted to transplantation without dental intervention (*n* = 22). Posttransplant complications were observed in 75% of patients in group I and 95.4% in group II. The impact of infectious outbreaks in the occurrence of posttransplant infections was not statistically significant, as well as correlations between decayed, impacted, and semierupted teeth, fever of unknown origin, mucositis, and the survival rate of patients with preexisting foci; however, the infectious foci were significant when associated with acute GVHD, mainly impacted teeth and periapical lesions. A higher rate of complications was found in group II, indicating the importance of evaluation and pretransplant dental work. It was concluded that dental treatment before HSCT should not be radical. Restorative and preventative techniques, however, must be individually adjusted for each patient.

Yamagata et al. [46] also conducted a prospective study of 41 patients who were undergoing HSCT using a conservative dental protocol. All patients were evaluated by clinical examination of the oral soft and hard tissues and, if necessary, radiographs were requested. Of the 41 patients, 36 required one or more dental interventions. The following diagnoses and procedures were performed: 101 carious lesions: 40 were restored and 61 were untreated; 5 pulpitis treated with endodontics; 10 teeth with apical periodontitis greater than 5 mm and 33 with less than 5 mm: 7 lesions were surgically removed, 5 teeth were endodontically treated (including two with symptomatology), and 31 teeth with lesions smaller than 5 mm and asymptomatic received no treatment; 94 teeth with periodontitis: 6 were extracted and 88 preserved, with survey monitoring and hygiene education; 21 partially erupted wisdom teeth: 3 presenting symptomatology were removed, the rest were untreated. All dental procedures were performed up to 10 days before HSCT, without change interruption or delay in the planning of the transplant. No patient had signs or symptoms of odontogenic infection during the immunosuppression period. The authors concluded that the conservative protocol appeared to be suitable for pre-HSCT patients.

Abdullah and Ahmad [47] conducted a study of 44 pediatric patients. Dental conditions were evaluated by clinical examination before HSCT. In the case of symptomatic tooth periapical radiographs were performed. Decayed teeth considered unviable were extracted, and the others were restored. In patients at high-risk of caries, sealant was applied. All patients received oral hygiene guidelines. The patients were evaluated after 1, 3, and 6 months after HSCT. Most patients (65.9%) needed some type of pre-HSCT dental treatment, having performed 101 restorations, 13 extractions, and 19 sealants. Within 6 months of monitoring, 10% of the patients who did not receive pre-HSCT dental treatment had odontogenic infection. No cases of odontogenic infection were observed in patients who previously received dental care. The authors concluded that the pre-HSCT dental treatment can reduce the occurrence of infection of dental origin, and it is important to prevent serious infections.

The US National Cancer Institute [48] points out that the time of reconstitution of the immune system in transplant patients can range from 6 to 12 months and that the dental care routine should not be done in this period, including scaling and periodontal planning. Procedures that produce aerosol, such as ultrasound equipment and high speed, can also present a risk of aspiration of debris and bacteria and cause pneumonia in these patients [19, 48]. If emergency treatment is required, strategies for reducing aerosol aspiration and antibiotic prophylaxis should be used. Finally, it is recommended that the use of IgG, antibiotics, corticosteroids, and/or platelet transfusion should be considered before implementing invasive procedures [48].

## 5. Final Considerations

From the literature review conducted, several oral manifestations in leukemic patients arose. These manifestations are often the first sign of leukemia and may present clinically as leukemic infiltration in oral tissues as well as simulating a periapical lesion. Other symptoms may occur such as pale mucosa, poor wound healing, bleeding (petechiae and ecchymoses), atypical or recurrent candidiasis, recurrent herpes infections, and ulcerations in the oral mucosa. During antineoplastic treatment (chemotherapy, mostly), the main complication is mucositis.

Other conditions that may also occur include bleeding, increase the rate of decay, infection, gum abscess, recurrent herpetic stomatitis, candidiasis, salivary gland dysfunction, xerostomia, dysgeusia, and pain. In the posttherapy period, patients are considered cured and usually present no sequelae of treatment.

Oral manifestations are similar in patients undergoing HSCT; however, generally these cases are due to long-term immunosuppression of the patient even after the transplantation. Special features are observed in patients undergoing allogeneic HSCT, such as cGVHD, which typically manifests as lichen-type features, hyperkeratotic plaques, mucocele, and fibrosis with limited mouth opening, and are more likely to develop malignancies such as squamous cell carcinoma.

Performing dental procedures can offer risk to the patient, depending on his state of health and phase of therapy. Furthermore, some procedures offer greater risk than others. Thus, noninvasive procedures (type I and type II) can be performed at any stage of the disease or treatment. Type III procedures may require special care. Finally, invasive procedures (types IV, V, and VI) offer higher risk. In emergency situations of risk considered, particularly those involving pain (acute cases), the patient should be assisted, if necessary, in a hospital setting, with the institution of measures to increase the hematological indices (transfusions) and, if applicable, with antibiotic coverage.

In assessing patients for dental procedures, two hematological indices are particularly important: neutrophil and platelet counts. At low levels of neutrophil counts, and when the procedure cannot be delayed, prophylactic antibiotic therapy protocols should be considered, being variable according to the degree of neutropenia; there is no strict consensus among authors, but most recommended antibiotic prophylaxis with values less than 1,000 cells/mm^3^. In the case of the platelet count, the authors consider the need for transfusion from indices between 40,000 and 60,000 cells/mm^3^.

Thus, we conclude, based on the literature review presented here, that the dental treatment in relation to haematological indices presented by patients with leukemia should follow some judicious protocols, mainly related to neutrophil and platelet counts. However, it is noteworthy that many of these studies are based on expert opinion. The presence of the dentist in a multidisciplinary team is essential, since we understand that maintaining oral health contributes significantly to the overall health and improved quality of life for patients through the use of dental approaches based on scientific evidence, preventive, curative, and palliative in nature.

## Figures and Tables

**Table 1:** Minimum haematological values for performance of invasive dental procedures in prechemotherapy treatment patients according to different authors.

Authors	Platelet count	Neutrophil count
Eversole et al., 2001 [33]	**<50,000** **c** **e** **l** **l**/**m** **m** ^3^: not perform dental or periodontal surgery in office setting.	—

Little et al., 2007 [5]	**<50,000** **c** **e** **l** **l**/**m** **m** ^3^: avoid invasive procedures. **<40,000** **c** **e** **l** **l**/**m** **m** ^3^: perform transfusions in invasive procedures.	**<500** **c** **e** **l** **l**/**m** **m** ^3^: antimicrobial prophylaxis (or with leukocytes <2,000 cells/mm^3^).

American Academy of Pediatric Dentistry, 2013 [1]	**>75,000** **c** **e** **l** **l**/**m** **m** ^3^: without additional support. **40,000 to 75,000** **c** **e** **l** **l**/**m** **m** ^3^: Platelet transfusion may be considered in the preoperative and postoperative (24 hours). **<40,000** **c** **e** **l** **l**/**m** **m** ^3^: Postpone the dental treatment. In the case of dental emergency, contact the patient's physician before dental treatment to discuss supportive measures, such as platelet transfusion, control of bleeding, and need for hospitalization. Other coagulation tests may be necessary in some cases.	**>1,000** **c** **e** **l** **l**/**m** **m** ^3^: no need for antibiotic prophylaxis. Some authors suggest that prophylaxis is performed with values between 1,000 and 2,000cell/mm^3^ (following recommendations of the American Heart Association). If infection is present or there is doubt, more aggressive antibiotic prophylaxis may be indicated and should be discussed with the medical team. **<1,000** **c** **e** **l** **l**/**m** **m** ^3^: Postpone the dental treatment. In cases of emergency, discuss antibiotic coverage and endocarditis prophylaxis before treatment with the medical team. Hospitalization may be required.

US National Cancer Institute, 2011 [2]	**>60,000** **c** **e** **l** **l**/**m** **m** ^3^: without additional support. **30,000 to 60,000** **c** **e** **l** **l**/**m** **m** ^3^: optional transfusion for noninvasive procedure. **<30,000** **c** **e** **l** **l**/**m** **m** ^3^: Platelets should be transfused 1 h before the procedure. Obtain immediate postinfusion platelet count; transfuse regularly to maintain counts >30,000–40,000 cell/mm^3^ until the start of healing.	**>2,000** **c** **e** **l** **l**/**m** **m** ^3^: without the need for antibiotic prophylaxis. **1,000 to 2,000** **c** **e** **l** **l**/**m** **m** ^3^: antibiotic prophylaxis (low risk). **<1,000** **c** **e** **l** **l**/**m** **m** ^3^: antibiotic prophylaxis with Amikacin 150** **mg/m^2^ 1** **h before surgery and Ticarcillin 75** **mg/Kg IV 1 h before surgery. Repeat both 6** **h postoperative.

**Table 2:** Minimum hematological values for performing invasive dental procedures in patients undergoing chemotherapy, according to different authors.

Authors	Platelet count	Neutrophil count
Sonis et al., 1995 [3]	**<100,000** **c** **e** **l** **l**/**m** **m** ^3^: elective dental treatment should be postponed.	**<3,500** **c** **e** **l** **l**/**m** **m** ^3^ (leukocytes): elective dental treatment should be postponed.
Haytac et al., 2004 [32]	**<40,000** **c** **e** **l** **l**/**m** **m** ^3^: periodontal probing and dental extractions contraindicated.	**<1,500** **c** **e** **l** **l**/**m** **m** ^3^: periodontal probing and dental extractions contraindicated.
Brennan et al., 2008 [22]	**<50,000** **c** **e** **l** **l**/**m** **m** ^3^: contraindication to perform invasive procedures.	**<1,000** **c** **e** **l** **l**/**m** **m** ^3^: contraindication to perform invasive procedures.
Koulocheris et al., 2009 [34]	**>60,000** **c** **e** **l** **l**/**m** **m** ^3^: acceptable for oral surgery.	**>1,000** **c** **e** **l** **l**/**m** **m** ^3^: acceptable for oral surgery.

**Table 3:** Possibility of dental procedures in the prechemotherapy phase.

Procedure	Considerations and restrictions	Time before the start of CT
Type I		
Exam		
Clinical Radiographic Hygiene instructions	No restrictions.	—
Molding	Elective procedure, postpone	—
Type II		
Simple restorations (ART) Prophylaxis and supragingival scaling	No restrictions.	—
Orthodontics	Elective treatment, postpone. Consider removing orthodontic appliances.	—
Type III		
More complex restorations	Solely for adequacy of the oral environment. Consider use of provisional restorative materials (e.g., glass ionomer).	—
Scaling and root planning (subgingival)	Invasive procedure of high-risk carried out carefully. To evaluate hematological indices of platelets and neutrophils. Need for antibiotic prophylaxis.	—
Endodontics		
Symptomatic tooth	Evaluate hematological indices of platelets and neutrophils. Need for antibiotic prophylaxis. Consider extraction if endodontics fail.	At least 1 week [1]
Asymptomatic tooth	Postpone (tricresol formalin) OR Evaluate hematological indices of platelets and neutrophils. Need for antibiotic prophylaxis.	At least 1 week [1]
Type IV		
Simple extractions	Invasive procedure of high-risk. Evaluate hematological indices of platelets and neutrophils. Need for antibiotic prophylaxis.	3 weeks; minimum 10–14 days [5] 2 weeks; minimum 7–10 days [1]
Curettage (gingivoplasty)	Elective procedure, invasive and high-risk. Postpone.	—
Type V		
Multiple extractions	If for adequacy of the oral environment, evaluate hematological indices of platelets and neutrophils. Need for antibiotic prophylaxis. If elective, postpone.	3 weeks; minimum 10–14 days [5] 2 weeks; minimum 7–10 days [1]
Flap surgery/gingivectomy Extraction of impacted tooth Apicoectomy Single implant placement	Elective procedure, invasive and high-risk. Postpone.	—
Type VI		
Extraction of an entire arch or both	If adequacy of the oral environment, evaluate hematological indices of platelets and neutrophils. Need for antibiotic prophylaxis. If elective, postpone.	3 weeks; minimum 10–14 days [5] 2 weeks; minimum 7–10 days [1]
Extraction of multiple impacted teeth Flap surgery Orthognathic surgery Placement of multiple implants	Elective procedure, invasive and high-risk. Postpone.	—

**Table 4:** Possibility of dental procedures in transchemotherapy phase.

Procedure	Considerations or restrictions	Time between cycles
Type I		
Exam		
Clinical Radiographic Hygiene instructions	No restrictions.	—
Molding	Elective procedure. Postpone.	—
Type II		
Simple restorations (ART) Prophylaxis and supragingival scaling	No restrictions.	—
Orthodontics	Elective treatment. Consider removing orthodontic appliances.	—
Type III		
More complex restorations	Solely for adequacy of the oral environment. Consider use of provisional restorative materials (eg., Glass ionomer).	—
Scaling and root planning (subgingival)	Invasive treatment, of high-risk, perform carefully. Evaluate hematological indices of platelets and neutrophils. Need for antibiotic prophylaxis.	—
Endodontics		
Symptomatic tooth	Evaluate hematological indices of platelets and neutrophils. Need for antibiotic prophylaxis. Consider extraction if endodontics fails.	At least 1 week [1]
Asymptomatic tooth	Postpone (tricresol formalin). OR Evaluate hematological indices of platelets and neutrophils. Need for antibiotic prophylaxis.	At least 1 week [1]
Type IV		
Simple extractions	Invasive treatment of high-risk. Evaluate hematological indices of platelets and neutrophils. Need for antibiotic prophylaxis.	3 weeks; minimum 10–14 days [5] 2 weeks; minimum 7–10 days [1]
Curettage (gingivoplasty)	Elective treatment, invasive and high-risk. Postpone.	—
Type V		
Multiple extractions	If adequacy of the oral environment, evaluate hematological indices of platelets and neutrophils. Need for antibiotic prophylaxis. If elective, postpone.	3 weeks; minimum 10–14 days [5] 2 weeks; minimum 7–10 days [1]
Flap surgery/gingivectomy Extraction of impacted tooth Apicoectomy Single implant placement	Elective procedure, invasive and high-risk. Postpone.	—
Type VI		
Extraction of an entire arch or both	If adequacy of the oral environment, evaluate hematological indices of platelets and neutrophils. Need for antibiotic prophylaxis. If elective, postpone.	3 weeks; minimum 10–14 days [5] 2 weeks; minimum 7–10 days [1]
Extraction of multiple impacted teeth Flap surgery Orthognathic surgery Placement of multiple implants	Elective procedure, invasive and high-risk. Postpone.	—

**Table 5:** Possibility of dental procedures in postchemotherapy phase.

Intervention in postchemotherapy	Considerations and restrictions
Type I	
Exam	
Clinic	No restrictions.
Radiographic	
Hygiene instructions	
Molding	
Type II	
Simple restorations (ART)	No restrictions.
Prophylaxis and supragingival cell scaling	
Orthodontics	Completed chemotherapy and after two years free of disease, one can restart the orthodontic treatment
Type III	
More complex restorations	No restrictions.
Scaling and root planning cell (subgingival)	
Endodontics	
Symptomatic tooth	
Asymptomatic tooth	
Type IV	
Simple extractions	Need for antibiotic prophylaxis until six months after completion of chemotherapy.
Curettage (gingivoplasty)	
Type V	
Multiple extractions	Need for antibiotic prophylaxis until six months after completion of chemotherapy.
Flap surgery/gingivectomy	
Extraction of impacted tooth	
Apicoectomy	
Single implant placement	
Type VI	
Extraction of an entire arch or both	Need for antibiotic prophylaxis until six months after completion of chemotherapy.
Extraction of multiple impacted cell teeth	
Flap surgery	
Orthognathic surgery	
Placement of multiple implants	

**Table 6:** Special considerations regarding oral complications, oral health, and dental treatment in pre-, immediate post-, and late post-HSCT.

Special considerations	Pre-HSCT (preconditioning)	Immediate post-HSCT (neutropenic conditioning phase and engraftment to hematopoietic recovery)	Late post-HCST (immune reconstitution/recovery from systemic toxicity and long-term survival)
Oral manifestations	(i) Oral infections (ii) Soreness (iii) Bleeding (iv) Temporomandibular dysfunction.	(i) Mucositis (ii) Dysgeusia (iii) Xerostomia (iv) Hemorrhage (v) Oral pain (vi) Opportunistic infections (vii) Neurotoxicity (viii) Temporomandibular dysfunction (ix) Acute GVHD.	(i) Chronic GVHD (ii) Late viral infections (iii) Salivary dysfunction (iv) Squamous cell carcinoma (v) Craniofacial growth abnormalities (children) (vi) Impairment of bones and teeth (children).

Oral health	(i) Identify and eliminate sources of existing or potential infection. (ii) Orientate the patient about the importance of maintaining oral health. (iii) Warn about the possible effects of antineoplastic therapy in the oral cavity.	(i) Maintain and reinforce the importance of optimal oral health. (ii) Treat side effects of HSCT therapy. (iii) Pay attention to periodontitis and gingivitis as potential sources of bacteremia.	(i) Diagnosis and treatment of mucosal lesions and lichen-type features with symptoms (ii) Caries prevention and reestablishment of oral health in case of rampant caries (iii) Treatment of hyposalivation and xerostomia (iv) Early detection of oral cancer and precursor lesions.

Dental treatment	(i) Complete necessary dental treatment (ii) Elective treatment should be delayed until the re-establishment of immunity (at least 100 days after transplant, or more in the case of oral complications or other cGVHD).	*Neutropenic conditioning phase* (i) Dental procedures should not be performed at this stage (ii) If emergencies, perform the necessary dental approach, with the participation of medical staff. *Engraftment to hematopoietic recovery* (i) Monitoring and management of oral complications of HSCT (ii) Invasive procedures only with the approval of the medical team (iii) Strengthening the maintenance guidelines of good oral hygiene and noncariogenic diet (iv) Special attention to xerostomia and GVHD.	*Immune reconstitution/recovery from systemic toxicity* (i) Periodic dental evaluation (ii) Avoid invasive procedures (iii) Clarify risks and benefits of orthodontic appliances. *Long-term survival* (i) Periodic dental evaluation (ii) In the first 12 months after HSCT: (a) avoid routine dental care, including scaling and periodontal planning; (b) if emergencies, strategies to reduce inhalation of aerosols and antibiotic prophylaxis; (c) before invasive procedures, consider the use of IgG, antibiotics, corticosteroids, and/or platelet transfusion.

**Table 7:** Possibility of dental procedures at various stages of chemotherapy.

Intervention	Pre	Trans	Post
Type I			
Exam			
Clinical	NR	NR	NR
Radiographic	NR	NR	NR
Oral hygiene instruction	NR	NR	NR
Molding	E	E	NR
Type II			
Simple restorations (ARTs)	NR	NR	NR
Prophylaxis and supragingival scaling	NR	NR	NR
Orthodontics	E	E	R
Type III			
More complex restorations	R	R	NR
Scaling and root planning (subgingival)	R HI, AP	R HI, AP	NR
Endodontics			
Symptomatic teeth	R HI, AP	R HI, AP	NR
Asymptomatic teeth	E, R HI, AP	E, R HI, AP	NR
Type IV			
Simple extractions	R, HI, AP	R, HI, AP	R
Curettage (gingivoplasty)	EIHR	EIHR	R
Type V			
Multiple extractions	R, HI, AP	R, HI, AP	R
Flap surgery/gingivectomy	EIHR	EIHR	R
Extraction of impacted tooth	EIHR	EIHR	R
Apicoectomy	EIHR	EIHR	R
Single implant placement	EIHR	EIHR	R
Type VI			
Extraction of an entire arch or both	R, HI, AP	R, HI, AP	R
Extraction of multiple impacted teeth	EIHR	EIHR	R
Flap surgery	EIHR	EIHR	R
Orthognathic surgery	EIHR	EIHR	R
Placement of multiple implants	EIHR	EIHR	R

NR: no restriction, R: with restriction, E: elective, EIHR: elective, invasive, and high-risk, HI: need for evaluation of hematological indices, and AP: antibiotic prophylaxis.
